# Dyssynchronous Ventricular Activation in Asymptomatic Wolff-Parkinson-White Syndrome: A Risk Factor for Development of Dilated Cardiomyopathy

**Published:** 2010-06-05

**Authors:** Floris EA Udink ten Cate, Nathalie Wiesner, Uwe Trieschmann, Markus Khalil, Narayanswami Sreeram

**Affiliations:** 1 Department of Paediatric Cardiology. University Hospital of Cologne, Germany; 2 Department of Anaesthesiology and Postoperative Intensive Care Medicine, University Hospital of Cologne, Cologne, Germany

**Keywords:** dilated cardiomyopathy, accessory pathway, Wolf-Parkinson-White syndrome, dyssynchrony, catheter ablation, speckle tracking imaging

## Abstract

A subset of children and adults with Wolff-Parkinson-White (WPW) syndrome develop dilated cardiomyopathy (DCM). Although DCM may occur in symptomatic WPW patients with sustained tachyarrhythmias, emerging evidence suggests that significant left ventricular dysfunction may arise in WPW in the absence of incessant tachyarrhythmias. An invariable electrophysiological feature in this non-tachyarrhythmia type of DCM is the presence of a right-sided septal or paraseptal accessory pathway. It is thought that premature ventricular activation over these accessory pathways induces septal wall motion abnormalities and ventricular dyssynchrony. LV dyssynchrony induces cellular and structural ventricular remodelling, which may have detrimental effects on cardiac performance. This review summarizes recent evidence for development of DCM in asymptomatic patients with WPW, discusses its pathogenesis, clinical presentation, management and treatment. The prognosis of accessory pathway-induced DCM is excellent. LV dysfunction reverses following catheter ablation of the accessory pathway, suggesting an association between DCM and ventricular preexcitation. Accessory pathway-induced DCM should be suspected in all patients presenting with heart failure and overt ventricular preexcitation, in whom no cause for their DCM can be found.

## Introduction

Clinically relevant arrhythmias are frequently encountered in patients with Wolff-Parkinson-White (WPW) syndrome  [1]. Reentrant supraventricular tachyarrhythmia, with orthodromic electrophysiological properties, is the most common arrhythmia in these patients [2]. A less common clinical manifestation in WPW is dilated cardiomyopathy (DCM). It has been recognized that recurrent and sustained tachyarrhythmia may induce dilated cardiomyopathy (tachyarrhythmia-induced cardiomyopathy) [3,4]. However, our group [5,6], and other investigators [7-12], have recently described another mechanism by which left ventricular (LV) dysfunction and DCM may occur in children and adults with overt ventricular preexcitation.

Two invariable clinical features associated with this type of DCM are: 1) absence of recurrent and sustained tachyarrhythmias ("asymptomatic" WPW), and 2) presence of right-sided accessory pathways, with overt ventricular preexcitation on 12-lead surface electrocardiogram (ECG) [5-12]. It is hypothesized that premature ventricular activation over this type of accessory pathway induces abnormal ventricular septal movement, LV dyssynchrony, LV remodelling and subsequently DCM [7-9]. This review summarizes the clinical and electrophysiologic evidence for this association, describes the electrophysiological properties of the accessory pathways, and discusses the management, treatment and clinical outcome of patients with "asymptomatic" WPW who develop LV dysfunction or DCM.

## Prevalence of DCM and LV dysfunction in WPW

The exact prevalence of DCM and LV dysfunction in patients with asymptomatic WPW is unknown. Only 27 cases of accessory pathway-induced DCM have been reported thus far [7-12]. The clinical and electrophysiological characteristics of these patients are summarized in Table 1. In our institution, we have seen 10 children with DCM and asymptomatic WPW during the last 10 years. However, it seems likely that this incidence has been underestimated. Approximately 27% of patients with WPW have a right-sided septally located accessory pathway [10], suggesting that some patients might have undergone an electrophysiological study (EPS) and catheter ablation before DCM developed. It is also possible that many patients have been incorrectly diagnosed to have idiopathic DCM.

LV dysfunction has been described in more than 50% of patients with right-sided septal accessory pathways [7,9]. Although prospective studies are lacking, small case series show that LV dysfunction in these patients may progress over time  [7-11]. It is hypothesized that newer echocardiographic techniques for regional and global myocardial function quantification, such as tissue Doppler imaging or Speckle Tracking echocardiography  [13], may demonstrate a much higher incidence of (subclinical) LV dysfunction in asymptomatic patients with right-sided septal accessory pathways.

## Pathophysiology

At present, the pathogenic mechanism for the development of LV dysfunction and DCM in patients with asymptomatic WPW has not yet been fully elucidated. Interestingly, a right-sided septal or paraseptal accessory pathway has been documented in all patients with overt ventricular preexcitation who developed LV dysfunction or DCM in the absence of recurrent tachyarrhythmias  [5-12]. This finding suggests that the location of the accessory pathway is an important feature in the pathogenesis of this disorder.

### Abnormal septal and ventricular wall motion in WPW

WPW is a disorder of normal cardiac conduction. It is characterized electrocardiographically by presystolic depolarization and contraction of a part of the ventricular myocardium via an accessory pathway. The effects of premature ventricular activation on cardiac wall motion and global ventricular function has intrigued cardiologists  [14-17]. Attempts have been made to study premature ventricular contraction patterns and wall motion using a variety of non-invasive modalities [14-17]. These studies have made several important observations with regard to normal and abnormal ventricular wall motion in WPW syndrome [14-20].

Left-sided accessory pathways may produce a typical premature anterior motion of the LV posterior wall, which can be identified on M-mode echocardiography as a small, distinct bump in early systole of the LV posterior wall [14]. On the other hand, right-sided accessory pathways may induce septal wall motion abnormalities, with similarities to those septal motion abnormalities seen in patients with left bundle branch block (LBBB) [21]. The abnormal septal motion pattern in patients with right-sided preexcitation consists of an early systolic posterior movement, a subsequent midsystolic anterior movement, with a delay in the usual late systolic septal movement. In the majority of these patients, the second posterior septal movement is interrupted by a prominent septal notch [15]. Although LV function was not measured in the earlier studies, it was found that the degree of abnormal ventricular contraction was dependent on the location of the accessory pathway in the heart  [14-17].

### Right sided accessory pathways and LV function

Recently, several investigators have demonstrated reduced systolic LV function in patients with right- sided septal and paraseptal accessory pathways [7,9]. A lower LV ejection fraction was noted in 56% of patients with septal and paraseptal accessory pathways [9]. In a study by Know et al. [7], systolic function was reduced in all patients with septal pathways, compared to patients with left or right-sided free wall pathways.

An important clinical finding is that cardiac function improves and normalizes in patients with LV dysfunction or DCM, following loss of ventricular preexcitation, either spontaneously or due to cardiac catheter ablation or medical therapy [5-12]. Moreover, several reports have shown an acute increase in LV systolic function directly following catheter ablation of the septal accessory pathway [5-7,9]. LV reverse remodelling and complete recovery of cardiac function occurs in the first weeks after loss of ventricular preexcitation [5-7,9], further supporting the pathogenic concept of accessory pathway-induced LV dysfunction and DCM.

### Detrimental effects of ventricular dyssynchrony on cardiac function

Septal accessory pathway-induced ventricular dyssynchrony is an important feature in the development of LV dysfunction and DCM [5,7,9]. We observed that the degree of dyssynchrony increased during follow-up in patients with asymptomatic WPW who subsequently developed DCM [5]. This pathogenetic concept is further supported by recent clinical and experimental work, demonstrating the detrimental effects of LV dyssynchrony on cardiac performance [22-25].

As timing is crucial for synchronous ventricular contraction and regional myocardial function, a delay in electrical activation of a myocardial segment, due to left bundle branch block (LBBB), right ventricular apical pacing, or chronic heart failure, may contribute to cardiac pump inefficiency [22-25]. Using tagged-MRI, Prinzen et al. [23] demonstrated in a canine model of LV dyssynchrony that systolic strain and wall stress, as well as external work are reduced in early-activated myocardial regions and increased in late-activated regions. This study explained why the energy generated by myocardial regions which are shorthening is absorbed by stretched regions, rather than resulting in propogation of blood or pressure generation. This change in strain and wall stress may result in wall motion abnormalities, myocardial perfusion defects, changes in coronary blood flow and regional molecular abnormalities, increased LV cavity volume, and asymmetrical changes in LV wall thickness, leading to reduced systolic cardiac function and heart failure  [22-25].

Two recent studies used conventional echocardiographic and speckle tracking imaging methods to quantify LV dyssynchrony in patients with WPW and septal accessory pathways [7,9]. Pre-ablation, most patients with septal accessory pathways showed significant LV dyssynchrony. Not surprisingly, LV dyssynchrony disappeared following catheter ablation (electrical resynchronization) with subsequent increase in systolic cardiac function [7,9]. It was further hypothesized that early septal activation induces dyskinetic/dyssynchronous septal motion, analogous to right ventricular apical pacing-induced LBBB [5,6,9,21]. This dyssynchronous septal myocardial segment may function much like an aneurysm. This so-called aneurysm may induce adverse LV remodelling with progressive dilation, with DCM as the most severe complication of septal accessory pathway-induced dyssynchrony [5-10]. These clinical studies further support the importance of dyssynchrony in the pathogenesis of LV dysfunction in patients with ventricular preexcitation.

However, not all patients with ventricular preexcitation and right-sided accessory pathways seem to be at risk for developing severe LV dysfunction or DCM. Possibly, the degree of pathway-induced LV dyssynchrony is an additional risk factor in these patients. Recent work suggests that different types of septal accessory pathways may induce a variable degree of LV dyssynchrony [5-7,9]. Interestingly, LV dysfunction was more pronounced in patients with septal and superoparaseptal pathways compared to patients with an inferoparaseptal pathway, probably due to a higher degree of pathway-mediated LV dyssynchrony [9]. However, this hypothesis warrants further prospective clinical studies.

## Clinical management and treatment

### Patients with WPW and DCM

As with all forms of heart failure and DCM in pediatric and adult patients, the underlying diagnosis must be considered. All patients should undergo standard electrocardiographic and echocardiographic assessment. Although there are certain age-related differences in the diagnostic work-up, the approach has to be individualized, depending on clinical findings, age and family history. A comprehensive summary of various important aspects in the diagnostic work-up of patients presenting with DCM have been well reviewed elsewhere [26,27]. In general, septal accessory pathway-induced DCM should be suspected in all patients presenting with DCM and overt ventricular preexcitation. This diagnosis is considered when other causes of DCM are excluded, particularly tachyarrhythmia-mediated DCM.

The prognosis of right-sided accessory pathway-induced DCM is excellent, when the diagnosis is suspected early in the clinical course and appropriate therapy is initiated [5-12]. The primary therapy for patients with asymptomatic WPW and accessory pathway-induced DCM is catheter ablation of the accessory pathway [1,2]. Cardiac catheter ablation has been proven an effective and safe therapy for patients with WPW-syndrome [1,2]. This approach is supported by the rapid normalization of cardiac function after loss of ventricular preexcitation.

Although catheter ablation is now considered as the standard therapy in adults and children > 12 kg with tachyarrhythmias [1,2,28], it may be associated with an unacceptable risk of complications in infants and small children. Due to these safety concerns, an alternative therapy may be offered in this pediatric age-group. Cadrin-Tourigny et al. described two small infants with septal accessory pathway-induced DCM who were successfully treated with amiodarone [10]. In both patients, pharmacological suppression of ventricular preexcitation was achieved within 3 months after amiodarone was started. LV function improved in both cases.

### Patients with WPW and LV dysfunction

**Figure 1:**
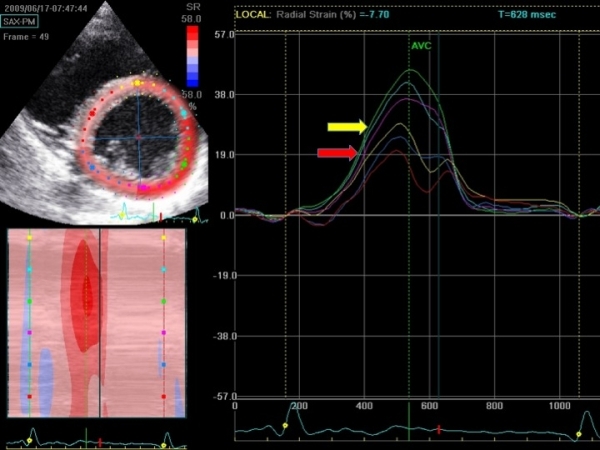
Radial strain assessment using 2D speckle tracking echocardiography. Radial two-dimensional strain in the parasternal short-axis view at the mid-ventricular level of a child with a septal accessory pathway and LV dysfunction. There is an inhomogeneous pattern of radial strain, with severely reduced peak radial strain values for septal myocardial segments (yellow and red arrows). Radial peak strain values are presented as positive values.

**Figure 2:**
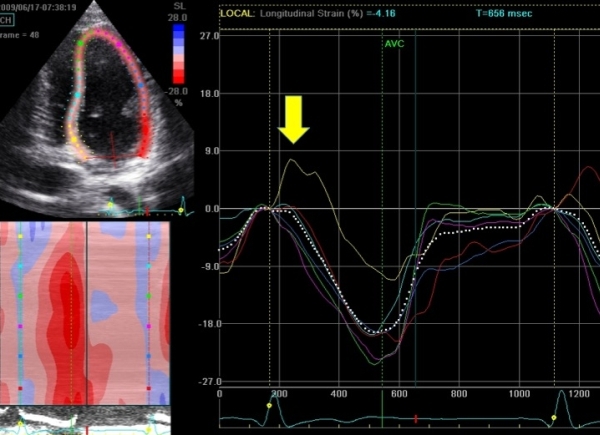
Longitudinal peak systolic strain values and curves of 6 myocardial segments obtained from a 4-chamber view in the same patient as in Figure 1. The septal basal segment (yellow arrow) shows reduced longitudinal peak systolic strain compared to the other segments. The septal basal segment stretches and relaxes in early systole, demonstrating regional systolic dysfunction. In addition, maximal peak systolic strain occurs after closure of the aortic valve (AVC). This part of the ventricle may function as an aneurysm during follow-up, inducing adverse remodelling and dyssynchrony (see text for further explanation).

Many asymptomatic patients with WPW and septal accessory pathways have LV dysfunction [7,9]. The extent of LV dysfunction can nowadays be accurately quantified using tissue Doppler imaging or 2D speckle tracking echocardiography [13]. In our institution, we use 2D speckle tracking imaging to quantify global and regional myocardial function, and degree of ventricular dyssynchrony. Figure 1 and 2 show an example of this approach in a child with asymptomatic WPW and LV dysfunction. Although the therapy of choice in these patients is not yet known, close echocardiographic monitoring of cardiac size and function is mandatory. It seems reasonable that catheter ablation should be reserved for those patients in whom progression of adverse LV remodelling, dyssynchrony and deteriorating cardiac function are demonstrated during follow-up.

Another interesting aspect in the clinical management of patients with WPW and LV dysfunction is whether QRS duration can be used for assessment of degree of ventricular dyssynchrony. Recent reports have shown an increased QRS duration in patients with septal and paraseptal accessory pathways [7,9]. However, this observation was not confirmed in our studies [5,6]. Therefore, QRS duration alone cannot identify all patients at risk for DCM. This may be particularly true for children, in whom intracardiac conduction velocities may be more rapid. Other non-invasive methods should be included in the risk stratification of these patients.

## Conclusions

Accessory pathway-induced DCM is a newly recognized form of LV dysfunction in asymptomatic WPW patients with right-sided pathways, in the absence of incessant tachyarrhythmias. Right-sided accessory pathways mediate ventricular dyssynchrony, inducing adverse remodelling and DCM. With loss of ventricular preexcitation, either spontaneously or after catheter ablation, LV function reverses completely. The prognosis of accessory pathway-induced DCM is excellent.

## Figures and Tables

**Table 1:**
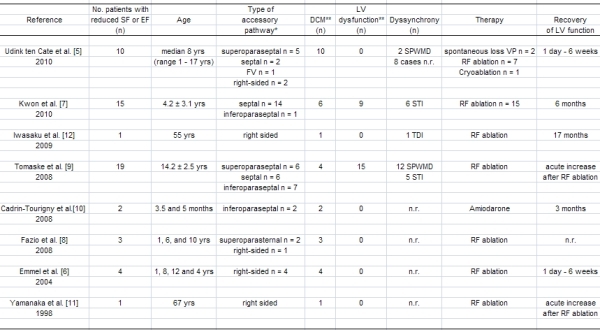
Summary of clinical and electrophysiological characteristics of all patients with Wolff-Parkinson-White and dilated cardiomyopathy or left ventricular dysfunction reported in the literature between 1998 and 2010

DCM = dilated cardiomyopathy; EF = ejection fraction; FV = fasciculoventricular fiber; LV = left ventricular; n.r. = not reported; RF = radiofrequency; SF = shortening fraction; SPWMD = septal-to-posterior wall motion delay; STI = speckle tracking imaging; TDI = tissue Doppler imaging; VP = ventricular preexcitation; yrs = years. *    Accessory pathways are described using the nomenclature presented by the Cardiac Nomenclature Study Group. In brief, the proposed terminology is based on anatomic positions, rather than depicting the position of the atrioventricular (AV) junctions [29]. Using this terminology, an anteroseptal or parahisian accessory pathway is referred to as superoparaseptal; an posteroseptal pathway as inferoparaseptal; and a midseptal accessory pathway as septal. **    DCM was defined as a LV end-diastolic dimension above the > 97th percentile of heart size corrected for weight with a shortening fraction of < 25% or ejection fraction < 45%  [5]. Patients who did not meet these criteria are grouped as LV dysfunction.
